# Evaluation of Cytokine Levels in Cardiac Transthyretin and Immunoglobulin Light Chain Amyloidosis and Their Correlation with Myocardial Inflammatory Cells and MACE

**DOI:** 10.3390/biomedicines13092254

**Published:** 2025-09-12

**Authors:** Nicolas Musigk, Phillip Suwalski, Maximilian Müller, Michele Violano, Karin Klingel, January Weiner, Dieter Beule, Ulf Landmesser, Bettina Heidecker

**Affiliations:** 1 Department of Cardiology, Angiology and Intensive Care Medicine, Deutsches Herzzentrum der Charité, Hindenburgdamm 30, 12203 Berlin, Germany; 2 Cardiopathology, Institute for Pathology and Neuropathology, University Hospital Tübingen, 72076 Tübingen, Germany; 3 Core Unit for Bioinformatics (CUBI), Berlin Institute of Health (BIH) at Charité—Universitätsmedizin Berlin, 10117 Berlin, Germany; 4 Berlin Institute of Health (BIH) at Charité—Universitätsmedizin Berlin, 10117 Berlin, Germany

**Keywords:** amyloidosis, myocarditis, heart failure, cardiomyopathy, cytokines, inflammation

## Abstract

**Aims:** Myocardial inflammation in cardiac amyloidosis is associated with poor clinical outcomes. This study aimed to (a) investigate the relationship between peripheral blood cytokine levels and the presence of inflammatory cells within the myocardium, and to (b) evaluate the potential of cytokines as predictors of major adverse cardiovascular events (MACE) in transthyretin (ATTR) and immunoglobulin light chain (AL) cardiac amyloidosis. **Methods:** Peripheral blood samples were collected from 50 patients with cardiac ATTR or AL amyloidosis between 2018 and 2023 at baseline and every three months during follow-up visits. Cytokine analysis was performed using Olink’s Proximity Extension Assay. For MACE prediction analysis, only patients with MACE occurring within ±14 days of a study visit were included (*n* = 16). Associations were evaluated using linear models. **Results:** No significant associations were identified between the EMB-confirmed myocardial presence of inflammatory cells and cytokine levels. There was a trend of weak-to-moderate associations between serial blood cytokine levels and MACE, albeit this was non-significant after adjustment for multiple testing (FDR): r^2^ = 0.28 for PON3 (*p* = 0.00075, FDR = 0.28), SIGLEC1 (*p* = 0.00077, FDR = 0.28), and IL-6 (*p* = 0.00086, FDR = 0.31). **Conclusions:** Peripheral blood cytokine levels were not reliable biomarkers for the myocardial presence of inflammatory cells. PON3, SIGLEC1, and IL-6 demonstrated a statistically non-significant trend of a weak-to-moderate association with MACE in cardiac amyloidosis. Since we recently demonstrated that amyloidosis with an inflammatory component is associated with poor outcomes, these additional findings underscore the need for alternative approaches to identify and manage inflammation in this patient population.

## 1. Introduction

Cardiac transthyretin (ATTR) and immunoglobulin light chain (AL) amyloidosis are both diseases characterized by the accumulation of misconfigured proteins, which can lead to infiltrative cardiomyopathy and heart failure [1]. With the availability of disease-modifying drugs such as tafamidis [2,3], vutrisiran [4], or the NI006-antibody [5], the interest in cardiac amyloidosis has been rapidly increasing in the scientific community [6,7,8].

Recent studies have highlighted that cardiomyopathies of various etiologies, including amyloidosis, are often accompanied by cardiac inflammation [9,10,11]. There is growing evidence to suggest that this inflammation may contribute to heart failure [9,11,12,13,14,15,16]. As such, understanding the inflammatory processes in cardiac amyloidosis is becoming an increasingly important research focus.

In a study of 50 patients with ATTR and AL cardiac amyloidosis, endomyocardial biopsies (EMBs) revealed that 42% of these patients exhibited myocardial infiltration by CD3+ T cells and CD68+ macrophages [17]. Since myocardial inflammation has been reported to be associated with poor clinical outcomes in ATTR and AL cardiac amyloidosis [10,11], it is critical to better understand the underlying inflammatory mechanisms. Specifically, it remains unclear whether inflammation is simply a byproduct of cardiac amyloidosis or whether it actively drives disease progression. Additionally, to advance personalized therapies in cardiac amyloidosis, it is essential to identify patients who may benefit from targeted anti-inflammatory treatments. We therefore aimed to (a) investigate the relationship between peripheral blood cytokine levels and the presence of inflammatory cells within the myocardium confirmed by EMB, and to (b) evaluate the potential of cytokines as predictors of MACE in ATTR and AL cardiac amyloidosis.

## 2. Materials and Methods

### 2.1. Study Design, Study Participants, Recruitment, and Pre-Analytics

To investigate the association between peripheral blood cytokine levels and the presence of inflammatory cells within the myocardium and MACE, ethylenediaminetetraacetic acid (EDTA) plasma samples from 50 patients with cardiac ATTR or AL amyloidosis were collected between the years 2018 and 2023 and stored in the biobank of the Deutsches Herzzentrum der Charité, Campus Benjamin Franklin. Blood was obtained from each patient at the baseline visit and during study visits every three months. Blood samples were collected in EDTA tubes, and EDTA plasma was separated from the samples by centrifugation and stored at −80 °C.

An EMB was only performed when clinically indicated [1]. In order to focus on samples from patients with a definitive diagnosis of myocardial inflammation, only patients for whom an EMB sample was available were included in the correlation analysis of peripheral cytokine levels with myocardial immune cell infiltration.

For analyzing whether peripheral blood cytokine levels predict MACE, only patients with MACE occurring within ±14 days of a study visit were included. MACE were defined as at least one of the following: all-cause mortality, cardiovascular mortality, hospitalization for heart failure, or acute decompensated heart failure.

### 2.2. Endomyocardial Biopsies

EMB samples were obtained during routine diagnostic workup as indicated by the European Society of Cardiology (ESC) position statement on the diagnosis and treatment of cardiac amyloidosis [1]. The EMB samples were sent to, and analyzed by, a specialized cardiopathologist. Samples were stained for CD3, CD20, and CD68.

### 2.3. Cytokine Analysis

For analyzing peripheral blood cytokine levels in EDTA plasma samples, we utilized the Proximity Extension Assay (PEA) by Olink. This method is based on two antibodies carrying unique oligonucleotide sequences that bind to the target proteins. The proximity of the two antibodies leads to hybridization of the oligonucleotide sequences that they carry and makes them accessible for deoxyribonucleic acid (DNA) polymerases, which extend the strands. The DNA strands can then be quantified by a real-time polymerase chain reaction (PCR).

Olink’s PEA technology empowers high-throughput and highly specific proteomics and is therefore a suitable approach for biomarker development [18]. We chose the Olink Explore 384—Inflammation panel for our analyses, enabling us to include 368 proteins associated with inflammation. While the panel is called “Olink Explore 384” based on its 384-plex capacity, it measures 368 inflammation-related proteins, with the remaining assay space used for internal controls and quality assurance. Supplemental File S1 lists all of the cytokines included in the Olink Explore 384—Inflammation panel.

Library preparation, randomization, quality control, and data processing were conducted using Olink’s standard processes (see https://www.olink.com for more information, accessed on 1 July 2025). Normalized protein expression (NPX) represents the data and provides a relative quantification of the samples within our patient cohorts.

### 2.4. Statistical Analysis

Statistical analysis was conducted using R (version 4.2.1). We used standard linear models to analyze the association between predictor and response variables. In the first analysis, the predictors were the myocardial presence of inflammatory cells, and the response variables were cytokine levels as NPX values. In the second analysis, cytokine levels as NPX values were used as predictors, and MACE were the response variables. For repeated measures analysis, we used the packages lme4 (v. 1.1) and lmerTest (v. 3.1). The obtained *p*-values were corrected for the false discovery rate (FDR) using the Benjamini–Hochberg method. For the analysis of baseline patient characteristics, categorical variables were summarized as counts and percentages and analyzed with the chi-square test. Continuous variables were presented as medians with interquartile ranges (IQR) and compared using the Mann–Whitney U test.

### 2.5. Ethics

This investigation conforms with the principles outlined in the Declaration of Helsinki [19]. Furthermore, this study was reviewed and approved by the ethics committee of Charité—Universitätsmedizin Berlin. All patients provided their written informed consent.

## 3. Results

### 3.1. Study Population

We enrolled 50 patients with cardiac ATTR or AL amyloidosis in this study (Table 1).

EMB samples were obtained from 27 of these 50 patients. In the remaining patients, a diagnosis of cardiac amyloidosis was established non-invasively, in line with ESC recommendations, using 99mTc-DPD scintigraphy together with serum/urine electrophoresis and immunofixation [1]. These 27 cases with an EMB-based diagnosis were used to investigate the relationship between peripheral blood cytokine levels and the presence of inflammatory cells within the myocardium. Among them, 10 patients who showed no signs of myocardial inflammation on immunohistology served as the control group for the first analysis. There were no significant differences in baseline characteristics between the cohorts in this first analysis, including type of amyloidosis, age, sex, N-terminal pro b-type natriuretic peptide (NT-proBNP), or creatinine (Table 1).

For analyzing whether peripheral blood cytokine levels predict MACE, only patients with MACE occurring within ±14 days of a study visit were included, leading to 16 patients being included in the second analysis. A total of 21 patients were included, for which no MACE were recorded. Therefore, these patients served as the control group for the second analysis. Although a significant difference in baseline creatinine levels was observed between the cohorts (1.3 mg/dL [1.0–1.6] vs. 1.2 mg/dL [1.1–1.4]; *p* = 0.049), this difference is not considered to be clinically relevant (Table 1).

### 3.2. Cytokine Levels and Their Association with the Myocardial Presence of Inflammatory Cells in Cardiac Amyloidosis

**Figure 1:**
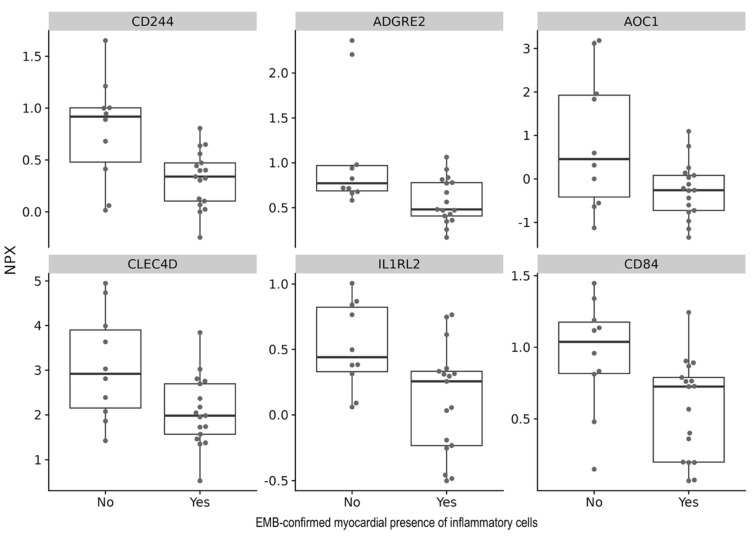
Relative protein levels of the top 6 cytokines associated with myocardial presence of inflammatory cells in cardiac ATTR and AL amyloidosis.

To analyze whether the EMB-verified presence of inflammatory cells within the myocardium causes changes in the levels of specific cytokines, we performed a differential analysis using a linear model. After correction for multiple testing, no cytokines were significantly associated with the EMB-verified presence of inflammatory cells within the myocardium (Figure 1, Table 2).

### 3.3. Cytokine Levels as Predictors of MACE in Cardiac Amyloidosis

**Figure 2:**
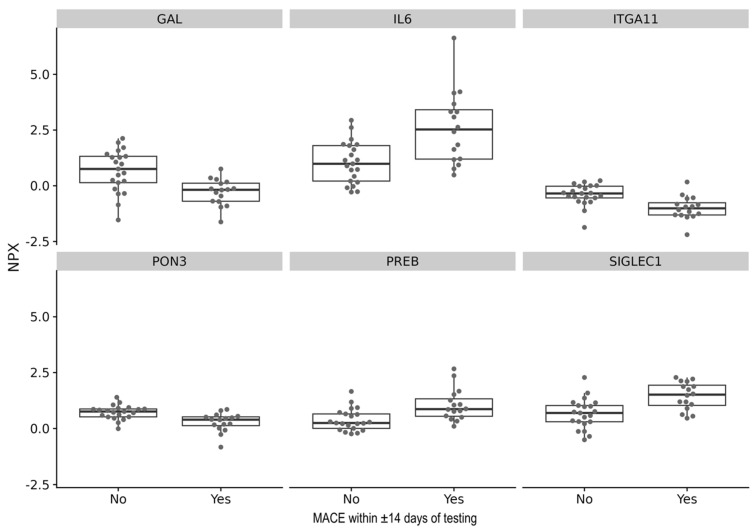
Relative protein levels of the top 6 cytokines associated with MACE in cardiac ATTR and AL amyloidosis.

A linear model was used to analyze whether cytokines can predict MACE within ±14 days of testing. Although there were no cytokines significantly associated with MACE after correction for multiple testing, there was a trend of a weak-to-moderate associations between serial blood cytokine levels of Paraoxonase 3 (PON3) (*p* = 0.00075, FDR = 0.28), Sialic-Acid-Binding Ig-Like Lectin 1 (SIGLEC1) (*p* = 0.00077, FDR = 0.28), and Interleukin 6 (IL-6) (*p* = 0.00086, FDR = 0.31) and MACE (Figure 2, Table 3).

## 4. Discussion

There is increasing evidence that myocardial inflammation in cardiac amyloidosis may play a relevant role for prognostic outcomes [10,11,17]. Therefore, we investigated the clinical implications of testing for myocardial inflammation by studying (a) the relationship between peripheral blood cytokine levels and the presence of inflammatory cells within the myocardium confirmed by EMB, and (b) the potential of cytokines as predictors of MACE in ATTR and AL cardiac amyloidosis. We hypothesized that cardiac background inflammation characterized by infiltration of inflammatory cells into the myocardium alters cytokine levels, serving as biomarkers for diagnosis, and that cytokines predict MACE in cardiac amyloidosis.

In our analysis, which may have been limited by the small sample size, no significant associations were found between serial blood cytokine levels and the status of myocardial immune cell infiltration. Given that we included all patients with myocardial inflammation, even those who did not meet the criteria outlined in the ESC position statement on myocarditis [20], it is also possible that, in cases of starker cardiac inflammation in amyloidosis, the effect on peripheral cytokine levels could be more pronounced.

The rationale behind our approach was the scientific discussion concerning to what extent the healthy heart contains immune cells and what the cut-off value is for defining cardiac inflammation in amyloidosis, and more generally, in cardiomyopathies caused by etiologies other than myocarditis [21]. Additionally, since an EMB provides only a snapshot in time, it remains unclear as to whether the biopsies from the patients of our cohort were taken during active inflammation or potentially a period of remission [9]. A key question in cardiac amyloidosis remains the timing and trajectory of inflammation, which also could have influenced our data.

Some studies suggest that during the chronic phase of myocardial inflammation, the overall density of immune cells within the myocardium decreases, while the proportion of T cells increases [9,22]. This may also influence peripheral cytokine levels, which might render them only an indirect measure of the status of myocardial inflammation at the time of assessment. We have previously demonstrated that in chronic myocarditis, inflammatory markers in the blood including C-reactive protein and troponin frequently normalize, while inflammation in the myocardium continues to progress [23].

This complexity in detecting myocardial inflammation is further compounded by the fact that an EMB can yield false negatives if the sample is taken from a region distant from areas of focal inflammation, as inflammation may not be uniformly distributed throughout the myocardium. In this regard, cardiac magnetic resonance (CMR) imaging, while considered the non-invasive gold standard in diagnosing myocarditis [24], might not be a suitable method in detecting myocardial inflammation in cardiac amyloidosis as the currently available data are limited and inconsistent [25]. Furthermore, it is known from myocarditis that CMR imaging has a limited diagnostic window of a few weeks for detecting myocardial inflammation [26], which may reduce its utility in the context of the chronic clinical trajectory of cardiac amyloidosis.

While our study may have been underpowered to detect robust associations for serial blood levels of cytokines and MACE, several cytokines, such as IL-6, demonstrated weak-to-moderate effect sizes and explained variance in relation to MACE. Nonetheless, none of these associations withstood false discovery rate (FDR) correction, underscoring the need for cautious interpretation. Importantly, while previous studies have reported associations between elevated IL-6 levels and adverse outcomes in amyloidosis [27,28], our findings may have been constrained by the limited sample size, emphasizing the need for validation in larger, adequately powered cohorts.

Previous studies using adult cardiomyocyte (AC16)-based models have demonstrated that AC16 cells treated with ATTR exhibit an enhanced response to pro-inflammatory cytokines and increased apoptosis [29]. Similarly, in AC16-based models of AL, there is an upregulation of IL-6 expression [30]. While interactions between these systems appear to influence clinical outcomes, they do not seem to be directly measurable in our cohort in terms of their immediate effect on MACE.

From a clinical perspective, evidence suggests that amyloidosis is associated with inflammation, which in turn leads to worse clinical outcomes [10,11]. More mechanistic studies are needed to stratify the role of inflammation in cardiac amyloidosis pathogenesis and the influence on the clinical trajectory of the patients, especially on the progression to heart failure. Furthermore, the insufficiency of cytokines in peripheral blood samples in assessing the status of cardiac inflammation in our cohort highlights the need for novel methods to detect cardiac inflammation and identify patients who might benefit from anti-inflammatory treatment. One promising tool could be the measurement of the cardiac magnetic field using magnetocardiography [31,32]. With the latest implementation of superconducting quantum interference device (SQUID) sensors, magnetocardiography detects myocardial alterations with increased sensitivity and has been successfully applied in inflammatory cardiomyopathy and cardiac amyloidosis research [32,33].

## Figures and Tables

**Table 1:** Patient characteristics.

	Analysis 1: Correlation of Cytokines with Myocardial Presence of Inflammatory Cells			Analysis 2: Correlation of Cytokines with MACE		
	EMB-Positive for Myocardial Presence of Inflammatory Cells (*n* = 17)	EMB-Negative for Myocardial Presence of Inflammatory Cells (*n* = 10)	*p*-Value	MACE ± 14 Days of a Study Visit (*n* = 16)	No MACE ± 14 Days of a Study Visit (*n* = 21)	*p*-Value
**ATTR** (*n*, %)	10 (58.8%)	7 (70.0%)	0.574	7 (43.8%)	15 (71.4%)	0.574
**AL** (*n*, %)	7 (41.2%)	3 (30.0%)		9 (56.2%)	6 (28.6%)	
**Age** in years (median, IQR)	77.0 (73.0–83.0)	76.5 (72.8–81.8)	1	78.0 (74.5–83.0)	78.0 (68.0–82.0)	0.684
**Sex female ** (*n*, %)	5 (29.4%)	2 (20.0%)	0.932	5 (31.3%)	4 (19.0%)	0.933
**BMI** in kg/m^2^ (median, IQR)	23.9 (22.6–25.3)	25.4 (22.4–26.7)	0.359	24.6 (21.3–27.3)	23.9 (21.8–25.9)	0.255
**hs-cTn** in ng/L (median, IQR)	69.0 (44.0–159.0)	55.0 (41.0–78.0)	0.590	81.5 (57.3–129.0)	57.0 (35.0–79.5)	0.182
**NT-proBNP** in ng/L (median, IQR)	5008.0 (2553.0–9448.0)	2112.5 (1163.3–5370.0)	0.204	6428.5 (3673.5–16,363.5)	2114.0 (1010.0–5008.0)	0.132
**Creatinine ** in mg/dL (median, IQR)	1.2 (1.1–1.4)	1.3 (1.1–1.5)	0.530	1.3 (1.0–1.6)	1.2 (1.1–1.4)	0.049

AL (immunoglobulin-light-chain amyloidosis), ATTR (transthyretin amyloidosis), BMI (body mass index), hs-cTn (high-sensitivity cardiac troponin), EMB (endomyocardial biopsy), IQR (interquartile range), MACE (major adverse cardiovascular event), NT-proBNP (N-terminal pro-B-type natriuretic peptide).

**Table 2:** Top 10 cytokines associated with myocardial presence of inflammatory cells in cardiac ATTR and AL amyloidosis.

Assay	Estimate	R^2^	*p*-Value	FDR
CD244	−0.47	0.28	0.0045	1
ADGRE2	−0.49	0.24	0.0101	1
AOC1	−1.13	0.22	0.0134	1
CLEC4D	−1.01	0.22	0.0142	1
IL1RL2	−0.41	0.22	0.0143	1
CD84	−0.37	0.21	0.0166	1
PRKAB1	−0.76	0.21	0.0167	1
OSCAR	−0.57	0.20	0.0207	1
RGS8	−1.04	0.18	0.0251	1
CD200	−0.31	0.18	0.0285	1

There was no significant difference in the expression of the top 10 cytokines between patients with and without inflammatory cells in the myocardium. The selected cytokines are the ten with the highest correlation to the myocardial presence of inflammatory cells, as determined by differential analysis using a linear model. ADGRE2 (Adhesion G-Protein-Coupled Receptor E2), AOC1 (Amine Oxidase Copper Containing 1), CD200 (Cluster of Differentiation 200), CD244 (Cluster of Differentiation 244, Natural Killer Cell Receptor 2B4), CD84 (Cluster of Differentiation 84), CLEC4D (C-Type Lectin Domain Family 4 Member D, Dectin-3), IL1RL2 (Interleukin 1 Receptor-Like 2, IL-36 Receptor), OSCAR (Osteoclast-Associated Immunoglobulin-Like Receptor), PRKAB1 (Protein Kinase AMP-Activated Non-Catalytic Subunit Beta 1), RGS8 (Regulator of G-Protein Signaling 8), Estimate (effect size estimate), FDR (false discovery rate calculated using Benjamini–Hochberg correction), r^2^ (coefficient of determination).

**Table 3:** Top 10 cytokines associated with MACE in cardiac ATTR and AL amyloidosis.

Assay	Estimate	R^2^	*p*-Value	FDR
PON3	−0.45	0.28	0.00075	0.28
SIGLEC1	0.79	0.28	0.00077	0.28
IL6	1.55	0.28	0.00086	0.31
ITGA11	−0.59	0.27	0.00107	0.39
GAL	−0.95	0.26	0.00136	0.49
PREB	0.66	0.24	0.00193	0.70
PRKCQ	0.73	0.24	0.00196	0.71
EPCAM	−0.81	0.23	0.00250	0.90
PNPT1	1.04	0.22	0.00320	1.00
TNFRSF13C	0.80	0.22	0.00330	1.00

There was no significant difference in the expression of the top 10 cytokines between patients with and without MACE within ±14 days of testing. The selected cytokines are the ten with the highest correlation to MACE ±14 days of testing, as determined by differential analysis using a linear model. EPCAM (Epithelial Cell Adhesion Molecule), GAL (Galanin and GMAP Prepropeptide), IL6 (Interleukin 6), ITGA11 (Integrin Subunit Alpha 11), PON3 (Paraoxonase 3), PNPT1 (Polyribonucleotide Nucleotidyltransferase 1), PREB (Prolactin Regulatory Element Binding), PRKCQ (Protein Kinase C Theta), SIGLEC1 (Sialic-Acid-Binding Ig-Like Lectin 1), TNFRSF13C (Tumor Necrosis Factor Receptor Superfamily Member 13C), Estimate (effect size estimate), FDR (false discovery rate calculated using Benjamini–Hochberg correction), r^2^ (coefficient of determination).

## Data Availability

Data presented in this study is contained within the article and Supplementary Material. Further inquiries can be directed to the corresponding author.

## Supplementary Materials

The following supporting information can be downloaded at: https://www.mdpi.com/article/10.3390/biomedicines13092254/s1, Table S1: Olink Explore 384—Inflammation panel.

## References

[1] Garcia-Pavia P., Rapezzi C., Adler Y., Arad M., Basso C., Brucato A., Burazor I., Caforio A.L.P., Damy T., Eriksson U. (2021). Diagnosis and treatment of cardiac amyloidosis: A position statement of the ESC Working Group on Myocardial and Pericardial Diseases. Eur. Heart J..

[2] Maurer M.S., Schwartz J.H., Gundapaneni B., Elliott P.M., Merlini G., Waddington-Cruz M., Kristen A.V., Grogan M., Witteles R., Damy T. (2018). Tafamidis Treatment for Patients with Transthyretin Amyloid Cardiomyopathy. N. Engl. J. Med..

[3] Rapezzi C., Elliott P., Damy T., Nativi-Nicolau J., Berk J.L., Velazquez E.J., Boman K., Gundapaneni B., Patterson T.A., Schwartz J.H. (2021). Efficacy of Tafamidis in Patients With Hereditary and Wild-Type Transthyretin Amyloid Cardiomyopathy: Further Analyses From ATTR-ACT. JACC Heart Fail..

[4] Fontana M., Berk J.L., Gillmore J.D., Witteles R.M., Grogan M., Drachman B., Damy T., Garcia-Pavia P., Taubel J., Solomon S.D. (2025). Vutrisiran in Patients with Transthyretin Amyloidosis with Cardiomyopathy. N. Engl. J. Med..

[5] Garcia-Pavia P., Aus dem Siepen F., Donal E., Lairez O., van der Meer P., Kristen A.V., Mercuri M.F., Michalon A., Frost R.J.A., Grimm J. (2023). Phase 1 Trial of Antibody NI006 for Depletion of Cardiac Transthyretin Amyloid. N. Engl. J. Med..

[6] Musigk N., Heidecker B. (2022). Transthyretin amyloidosis: The picture is getting clearer. Eur. J. Heart Fail..

[7] Rubin J., Maurer M.S. (2020). Cardiac Amyloidosis: Overlooked, Underappreciated, and Treatable. Annu. Rev. Med..

[8] Müller M.L., Butler J., Heidecker B. (2020). Emerging therapies in transthyretin amyloidosis—A new wave of hope after years of stagnancy?. Eur. J. Heart Fail..

[9] Musigk N., Suwalski P., Golpour A., Fairweather D., Klingel K., Martin P., Frustaci A., Cooper L.T., Lüscher T.F., Landmesser U. (2024). The inflammatory spectrum of cardiomyopathies. Front. Cardiovasc. Med..

[10] Siegismund C.S., Escher F., Lassner D., Kühl U., Gross U., Fruhwald F., Wenzel P., Münzel T., Frey N., Linke R.P. (2018). Intramyocardial inflammation predicts adverse outcome in patients with cardiac AL amyloidosis. Eur. J. Heart Fail..

[11] Müller M.L., Brand A., Mattig I., Spethmann S., Messroghli D., Hahn K., Violano M., Mitchell J.D., Hare J.M., Frustaci A. (2025). Myocardial Inflammation in Cardiac Transthyretin Amyloidosis: Prevalence and Potential Prognostic Implications. Circ. Heart Fail..

[12] Levine B., Kalman J., Mayer L., Fillit H.M., Packer M. (1990). Elevated circulating levels of tumor necrosis factor in severe chronic heart failure. N. Engl. J. Med..

[13] Mann D.L. (2015). Innate immunity and the failing heart: The cytokine hypothesis revisited. Circ. Res..

[14] Adamo L., Rocha-Resende C., Prabhu S.D., Mann D.L. (2020). Reappraising the role of inflammation in heart failure. Nat. Rev. Cardiol..

[15] Pieske B., Tschöpe C., de Boer R.A., Fraser A.G., Anker S.D., Donal E., Edelmann F., Fu M., Guazzi M., Lam C.S.P. (2019). How to diagnose heart failure with preserved ejection fraction: The HFA–PEFF diagnostic algorithm: A consensus recommendation from the Heart Failure Association (HFA) of the European Society of Cardiology (ESC). Eur. Heart J..

[16] Van Linthout S., Tschöpe C. (2017). Inflammation-Cause or Consequence of Heart Failure or Both?. Curr. Heart Fail. Rep..

[17] Klingel K., Hehn A., Sauter M. (2017). P5403The impact of inflammation on the outcome of cardiac amyloidosis. Eur. Heart J..

[18] Lundberg M., Eriksson A., Tran B., Assarsson E., Fredriksson S. (2011). Homogeneous antibody-based proximity extension assays provide sensitive and specific detection of low-abundant proteins in human blood. Nucleic Acids Res..

[19] Rickham P.P. (1964). HUMAN EXPERIMENTATION. CODE OF ETHICS OF THE WORLD MEDICAL ASSOCIATION. DECLARATION OF HELSINKI. Br. Med. J..

[20] Caforio A.L., Pankuweit S., Arbustini E., Basso C., Gimeno-Blanes J., Felix S.B., Fu M., Heliö T., Heymans S., Jahns R. (2013). Current state of knowledge on aetiology, diagnosis, management, and therapy of myocarditis: A position statement of the European Society of Cardiology Working Group on Myocardial and Pericardial Diseases. Eur. Heart J..

[21] Halushka M.K., d’Amati G., Bois M.C., De Gaspari M., Giordano C., Klingel K., Leduc C., Ohta-Ogo K., Ozcan I., Rizzo S. (2025). The frequency of CD3+ lymphocytes in non-myocarditis endomyocardial biopsies. Cardiovasc. Pathol..

[22] Fairweather D., Kaya Z., Shellam G.R., Lawson C.M., Rose N.R. (2001). From infection to autoimmunity. J. Autoimmun..

[23] Berg J., Kottwitz J., Baltensperger N., Kissel C.K., Lovrinovic M., Mehra T., Scherff F., Schmied C., Templin C., Lüscher T.F. (2017). Cardiac Magnetic Resonance Imaging in Myocarditis Reveals Persistent Disease Activity Despite Normalization of Cardiac Enzymes and Inflammatory Parameters at 3-Month Follow-Up. Circ. Heart Fail..

[24] Schulz-Menger J., Collini V., Gröschel J., Adler Y., Brucato A., Christian V., Ferreira V.M., Gandjbakhch E., Heidecker B., Kerneis M. (2025). 2025 ESC Guidelines for the management of myocarditis and pericarditis: Developed by the task force for the management of myocarditis and pericarditis of the European Society of Cardiology (ESC)Endorsed by the Association for European Paediatric and Congenital Cardiology (AEPC) and the European Association for Cardio-Thoracic Surgery (EACTS). Eur. Heart J..

[25] Kottam A., Hanneman K., Schenone A., Daubert M.A., Sidhu G.D., Gropler R.J., Garcia M.J., on behalf of the American Heart Association Council on Cardiovascular Radiology and Intervention (2023). State-of-the-Art Imaging of Infiltrative Cardiomyopathies: A Scientific Statement From the American Heart Association. Circ. Cardiovasc. Imaging.

[26] Lurz P., Luecke C., Eitel I., Föhrenbach F., Frank C., Grothoff M., Waha S.d., Rommel K.-P., Lurz J.A., Klingel K. (2016). Comprehensive Cardiac Magnetic Resonance Imaging in Patients With Suspected Myocarditis. JACC.

[27] Hein S.J., Knoll M., Aus dem Siepen F., Furkel J., Schoenland S., Hegenbart U., Katus H.A., Kristen A.V., Konstandin M. (2021). Elevated interleukin-6 levels are associated with impaired outcome in cardiac transthyretin amyloidosis. World J. Cardiol..

[28] Lugitsch J., Hoeller V., Schwegel N., Santner V., Gollmer J., Kolesnik E., Lipp R., Niedrist T., Rainer P., Zirlik A. (2023). Interleukin-6 plasma levels predict mortality and heart failure events in cardiac transthyretin amyloidosis. Eur. Heart J..

[29] Ghosh S., Villacorta-Martin C., Lindstrom-Vautrin J., Kenney D., Golden C.S., Edwards C.V., Sanchorawala V., Connors L.H., Giadone R.M., Murphy G.J. (2023). Mapping cellular response to destabilized transthyretin reveals cell- and amyloidogenic protein-specific signatures. Amyloid.

[30] Jordan T.L., Maar K., Redhage K.R., Misra P., Blancas-Mejia L.M., Dick C.J., Wall J.S., Williams A., Dietz A.B., van Wijnen A.J. (2020). Light chain amyloidosis induced inflammatory changes in cardiomyocytes and adipose-derived mesenchymal stromal cells. Leukemia.

[31] Golpour A., Suwalski P., Landmesser U., Heidecker B. (2023). Case report: Magnetocardiography as a potential method of therapy monitoring in amyloidosis. Front. Cardiovasc. Med..

[32] Brala D., Thevathasan T., Grahl S., Barrow S., Violano M., Bergs H., Golpour A., Suwalski P., Poller W., Skurk C. (2023). Application of Magnetocardiography to Screen for Inflammatory Cardiomyopathy and Monitor Treatment Response. J. Am. Heart Assoc..

[33] Müller M.L., Suwalski P., Wilke F., Latinova E., Satilmis G., Musigk N., Brand A., Mattig I., Knebel F., Messroghli D. (2025). Magnetocardiography for the non-invasive diagnosis of myocardial inflammation in cardiac amyloidosis: A proof-of-concept study. ESC Heart Fail..

